# Application of Electrophysiology in Non-Macular Inherited Retinal Dystrophies

**DOI:** 10.3390/jcm12216953

**Published:** 2023-11-06

**Authors:** Yulia Haraguchi, Tsun-Kang Chiang, Minzhong Yu

**Affiliations:** Department of Ophthalmology, University Hospitals, Case Western Reserve University, Cleveland, OH 44106, USA

**Keywords:** electrophysiology, multifocal electroretinogram, full-field electroretinogram, electrooculogram, pattern electroretinogram, visual evoked potential, retina, inherited retinal dystrophies, retinitis pigmentosa, progressive cone and cone-rod dystrophy, bradyopsia, Bietti crystalline dystrophy, late-onset retinal degeneration, fundus albipunctatus

## Abstract

Inherited retinal dystrophies encompass a diverse group of disorders affecting the structure and function of the retina, leading to progressive visual impairment and, in severe cases, blindness. Electrophysiology testing has emerged as a valuable tool in assessing and diagnosing those conditions, offering insights into the function of different parts of the visual pathway from retina to visual cortex and aiding in disease classification. This review provides an overview of the application of electrophysiology testing in the non-macular inherited retinal dystrophies focusing on both common and rare variants, including retinitis pigmentosa, progressive cone and cone-rod dystrophy, bradyopsia, Bietti crystalline dystrophy, late-onset retinal degeneration, and fundus albipunctatus. The different applications and limitations of electrophysiology techniques, including multifocal electroretinogram (mfERG), full-field ERG (ffERG), electrooculogram (EOG), pattern electroretinogram (PERG), and visual evoked potential (VEP), in the diagnosis and management of these distinctive phenotypes are discussed. The potential for electrophysiology testing to allow for further understanding of these diseases and the possibility of using these tests for early detection, prognosis prediction, and therapeutic monitoring in the future is reviewed.

## 1. Introduction

Inherited retinal dystrophies (IRDs) encompass a diverse group of disorders affecting the structure and function of the retina, leading to progressive visual impairment and, in severe cases, blindness [1,2,3,4]. Inherited retinal diseases are classified into four categories, including rod-cone dystrophy, cone-rod dystrophy, chorioretinal degenerations and inherited macular dystrophies. The genetic and clinical heterogeneity of these rare disorders can make diagnosis and prognosis challenging, requiring full ophthalmologic evaluation, genetic testing, and electrophysiological testing. Advances in imaging modalities such as optical coherence tomography (OCT) and fundus autofluorescence (FAF) have detected subtle changes on the fundus exams, allowing for accurate characterizations of IRDs. Electrophysiological tests, including multifocal electroretinogram (mfERG), full-field ERG (ffERG), electrooculogram (EOG), pattern electroretinogram (PERG), and visual evoked potential (VEP), can help characterize IRDs by identifying the site of damage but also the cell type involved in the visual degenerative process.

Full-field ERG, under standardized recording protocols of International Society for Clinical Electrophysiology of Vision (ISCEV), is a widely used assessment for generalized retinal function that measures mass electrophysiological response to brief flashes of light [5]. A Ganzfeld (full-field) bowl is used to uniformly illuminate the entire retina, which evokes responses that are recorded by an electrode in contact with cornea, bulbar conjunctiva, or lower eyelid skin, with a reference electrode at the outer canthus and a ground electrode at forehead or an earlobe. Six different responses exist in assessing different parts of the visual pathway, based on whether in dark-adapted (DA) and light-adapted (LA) conditions and the flash strength in candela-seconds per square meter (cd·s·m^−2^) [6]. The three DA ERGs include responses to flash strengths of 0.01, 3, and 10 phot cd·s·m^−2^ (DA 0.01; DA 3; DA 10). DA 0.01 ERG arises from ON-bipolar cells of the rod system, eliciting only a b-wave and no detectable a-wave. DA 3.0 and 10.0 are mixed rod and cone responses, with greater rod system contribution in healthy retinas. The oscillatory potentials (OPs) response reflects the interactions between bipolar cells, amacrine cells, and ganglion cells. It has been reported that rod bipolar cell-AII/A17 amacrine cell reciprocal synapses are the source of OPs in mice [7]. We conclude that the reciprocal synapses between RBC and AII/A17 are the basis of the ERG OP oscillations of the light response. The two LA ERGs are the responses to a flash strength of 3 photcd·s·m^−2^, superimposed on a light-adapting background (luminance 30 cd·m^−2^) as single flashes (LA 3 ERG) and to 30 Hz flicker (LA 30-Hz ERG), respectively, in which a delayed time response suggests generalized cone dysfunction and reduced amplitude with normal peak time and is indicative of restricted cone dysfunction.

Multifocal electroretinogram (mfERG) usually employs a stimulus array of 61 or 103 hexagons that elicits initial negative deflection (N1) followed by a positive peak (P1) and negative peak (P2) from 61 or 103 corresponding retinal locations that can also be averaged in six concentric rings around the foveola [8,9] and in four quadrants. The mfERG has also been used to assess the loss of function in the macula and peripheral retinal regions and to detect cone-mediated responses in regions with remaining photoreceptor function [10]. Pattern electroretinogram (PERG) is usually recorded using an alternating black and white checkerboard pattern [11]. The responses from PERG include a positive P50 peak, which indicates macular function, and a negative N95 deflection, which reflects retinal ganglion cell function [12].

In addition to clinical assessments such as visual acuity, contrast sensitivity test, color vision test, and visual field test, electrophysiological testing provides a unique and objective evaluation of retinal function. Combining electrophysiological testing with other imaging modalities, such as OCT, provides useful information for diagnosis and characterization of disease, potentially in patients that are pre-symptomatic or have dysfunction or degeneration in isolated regions of the retina. In this review, we summarize the specific findings of different non-macular IRDs in electrophysiology and provide an overview of the different applications and limitations of electrophysiology testing in the diagnosis and management of commonly seen IRDs, listed in Table 1. We describe the clinical manifestation and genetic approach, followed by a review of the application of electrophysiological testing in the diagnosis and workup for each specific non-macular and non-choroid IRD. Inherited macular dystrophies and chorioretinal degenerations were excluded from this review, as macular IRDs were described extensively in our previous study [13] and the scope of the choroid degenerations lends itself to a separate, future review.

## 2. Application of Electrophysiology in Inherited Dystrophies

### 2.1. Retinitis Pigmentosa

Retinitis pigmentosa (RP) is the most common inherited retinal dystrophy and major cause of visual disability, with a worldwide prevalence of 1 in 4000 [36,37]. RP is characterized by rod photoreceptor degeneration associated with initial symptoms of reduced night vision (nyctalopia) and difficulty with dark adaptation, followed by progressive cone photoreceptor and RPE degeneration, which is associated with progressive loss of the visual field [38].

The clinical presentation of RP includes night blindness, tunnel vision, and gradual loss of central vision that can differ based on the large number of genes and alleles involved [38]. Classically, symptomatic visual loss in RP begins in adolescence, although the exact age of onset can widely differ by subtype [38]. Most RP cases are non-syndromic, meaning that the visual symptoms are isolated, while 20–30% of patients with RP are syndromic and associated with a non-ocular condition.

Non-syndromic retinitis pigmentosa is a group of genetically heterogeneous disorders that includes autosomal dominant (AD: 15–25% of cases), autosomal recessive (AR: 5–20%), X-linked (5–15%), and simplex forms (40–50%), with more than 70 genes and 300 mutations implicated [39]. About 26 gene mutations have been identified for the AD form of RP, a subtype characterized by decreased severity, slow progression, and later age of onset with the best long-term prognosis of preserving central vision [39,40]. Common genes implicated in autosomal dominant RP include the *RHO* gene (20–30% of AD RP cases), which encodes for rhodopsin and can lead to gain-of-function mutation in which large amounts of misfolded rhodopsin overwhelm the ubiquitin proteasome system and leads to cell toxicity [41]; the *PRPF31* gene (10%), a pre-mRNA slicing factor producing high levels of alternatively spliced transcripts in photoreceptors [41]; and the *RP1* gene (10%), a microtubule-associated protein in the outer segment disc [40]. X-linked RP tends to be one of the most severe forms of RP, with nyctalopia in childhood, followed by continued reduction in the visual fields until severe decrease in visual acuity (less than 20/200) occurs by the fourth decade of life [42]. The most common genes associated with X-linked RP include a non-sense mutation of the *RPGR* gene, which regulates photoreceptor cilia function, and the *RP2* gene, which is involved in protein transport in photoreceptor cells [14]. Most of the genes associated with autosomal recessive RP are rare and cause only 1% of all cases. Autosomal recessive mutations in the *PDE6* (phosphodiesterase) complex can be associated with early-onset RP due to its negative effects on rod phototransduction and intracellular cGMP levels [39]. Isolated cases are characterized by individuals who do not have affected family members and display clinical heterogeneity [15].

Around 20–30% of patients with RP are syndromic and associated with extra-ocular abnormalities [38]. Bardet–Biedl syndrome (BBS) and the Usher syndrome (USH) are the most frequent types of syndromic RP worldwide [16]. Bardet–Biedl syndrome is a rare autosomal recessive disease characterized by onset of retinal dystrophy with early macular development in the first decade of life and legal blindness in most patients by the second or third decade. Other characteristics of BBS include obesity, hypogonadism, developmental delay, cognitive deficits, renal abnormalities, and post-axial polydactyly [43]. USH syndrome is an autosomal recessive disease characterized by severe hearing loss and vestibular dysfunction, as well as an early onset of classical symptoms of *RP* around the first decade of life [44].

The diversity of pathogenesis of genetic defects associated with RP poses significant challenges in preventing the onset and advancement of the condition and in restoring vision. There is no current pharmacological therapy that has been clearly proven for efficacy, although some neuroprotective substances such as neurotrophic factors, Vitamin A and other antioxidants may delay photoreceptor and RPE cell loss [45]. Additionally, gene therapy, stem/retinal cell transplantation, and visual cortex prosthesis have been in development in recent decades [45,46]. The most successful progress in the treatment in recent years is the *RPE65* gene therapy that has been successfully used in clinical patients [47,48,49,50,51,52,53,54,55,56,57,58,59].

On fundus examination, the classic triad of features of RP includes bone spicule pigmentation, attenuation of retinal vessels, and waxy pallor of the optic nerve [38]. Bone-spicule pigmentation occurs due to retinal pigment epithelium (RPE) detachment and migration from Bruch’s membrane following photoreceptor degeneration, leading to pigmentary clumping around intraretinal vessels [60]. The optic nerve head may show waxy pallor in early disease due to hyperemia and may progress to significant pallor in advanced disease due to axon loss and glial cell response [60,61]. There are also optical coherence tomography (OCT)-detected structural changes, including disruption and loss of the ellipsoid zone, loss of choroidal vasculature, cystoid macular edema, and decreased retinal thickness [62,63,64].

**Figure 1:**
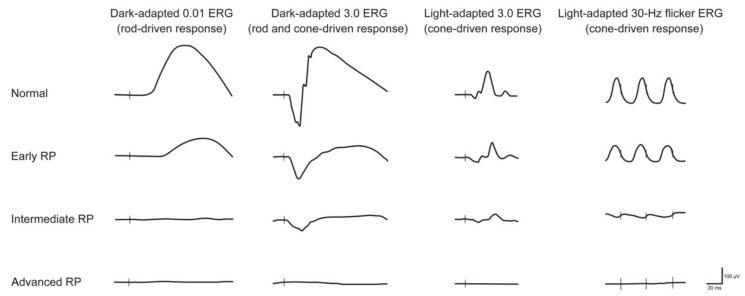
Electroretiongram (ERG) recordings in early, intermediate and advanced stages of retinitis pigmentosa (RP). Vertical lines indicate the moment of stimulus flash. As the RP progresses, the amplitude of responses decreases, and the implicit time may increase. Cone dysfunction typically lags behind the onset of rod dysfunction. Eventually, the ERG—under both scotopic and photopic conditions—is extinguished (reused from Verbakel et al., 2009 with permission [38]).

Use of electrophysiology in the diagnostic workup for RP can be valuable in the early detection and characterization of disease. The full-field electroretinogram (ffERG) may detect the early characteristic photoreceptor degeneration, even before symptoms are noticeable for patients, making it useful for the diagnosis of RP [36]. Full-field ERG recording enables the distinction between predominantly rod or cone system dysfunction (DA or LA protocols, respectively) and between generalized outer and inner retinal dysfunction. As seen in Figure 1, the ffERGs of RP patients characteristically include significantly decreased ERG amplitudes and increased latency compared to normal eyes in five ffERG protocols (DA 0.01, DA 10.0, OP, LA 3.0 and LA 3.0 30 Hz flicker) [65,66]. The LA 30 Hz, which objectively assesses cone system sensitivity, also correlates strongly with reduced visual acuity in RP patients [67]. These electrophysiologic findings correlate with clinical symptoms of nyctalopia and constriction of peripheral visual fields (rod photoreceptor dysfunction) and reduction in central vision (progressive cone photoreceptor dysfunction). Additionally, the location of the lesion affects the signal response to different extents. For example, the amplitudes of responses of rod, rod-cone, and cone [68] of typical RP patients with peripheral lesions gradually extending to the central retina are lower than those of pericentral RP patients with lesions at the major temporal arcade on the retina that relatively spare the far periphery [69].

In RP patients without visual acuity deterioration, the N1 and N1P1 mfERG components showed preserved central retinal function but were severely reduced outside zone 2 of the retina [70]. In RP, the mfERG delayed latency and decreased amplitude compared to controls has been shown to correspond with the regions of visual field losses [71]. The decreased amplitude of the central segment of mfERGs and increased latency of mfERG in the central retina have significant correlations to the loss of visual acuity [72]. Additionally, focal macular electroretinography amplitudes are reduced before the EZ thinning on OCT in the early stage of RP, which indicates that the focal macular ERG can be an earlier and reliable indicator of macular dysfunction [73]. However, mfERGs, like PERGs, may not be recommended as a primary outcome measure in patients with advanced RP and nonrecordable ffERG because the response in all tested areas can be very low [74]. On the electrooculogram (EOG), which measures the RPE potential in response to dark and light conditions, advanced RP is associated with a reduced light peak (Lp) to dark trough (Dt) ratio (i.e., Arden ratio) [62,75,76,77].

**Figure 2:**
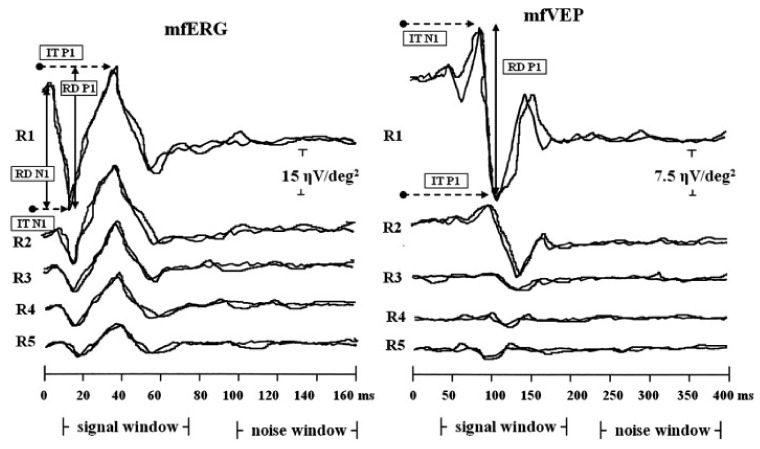
Examples of multifocal electroretinogram (mfERG) and multifocal visually evoked potential (mfVEP) recorded in one representative retinitis pigmentosa study eye. MfERG and mfVEP local responses were averaged in five retinal areas located at various degrees of eccentricity from the fovea: 0–2.5 (R1), 2.5–5 (R2), 5–10 (R3), 10–15 (R4) and 15–20 (R5) degrees. IT, implicit time; RAD, response amplitude density (reused from Parisi et al., 2009 with permission [10]).

The combination of mfERG and multifocal visually evoked potential technique (mfVEP) helps determine if there is an impact of regional retinal function on the visually evoked cortical activity, as VEPs are generated primarily in the visual cortex and may be affected by abnormalities along the visual pathway [78]. As seen in Figure 2, significant positive correlations were found between the mfERG response amplitude density (RAD) and the mfVEP RAD, as well as between the mfERG implicit time and the mfVEP implicit time, indicating that some of the mfERG responses were quantitatively correlated with mfVEP response components at corresponding retinal locations [10]. The functional measures (mfERGs) in RP usually confirm the preserved central retinal function with severely reduced function outside of the central retina, which may not be reflected by ffERGs [79].

Unlike ffERG, which represents a mass response of the entire retina for the rod and cone systems, the pattern electroretinogram (PERG) provides a measure of central retinal function and shows abnormal values with affected visual acuity [80]. PERG evokes retinal response with two components, the positive P50 and the negative N95, by a high-contrast checkerboard reversal pattern stimulus [11]. The PERG of RP patients show significantly lower P50 and N95 amplitudes, which represent macular photoreceptor and ganglion cell activity, respectively [81,82] and correlate with previous histological studies that showed significant reduction in the number of macular ganglion cells in RP [83,84]. Additionally, reductions in P50 amplitudes are associated with both reduced visual acuity and the reduced length of ellipsoid layer on OCT, providing objective measures that may assist in monitoring disease progression [67]. Pattern visual evoked potentials (PVEPs) of RP patients show characteristic N75, P100 and N135 peaks with smaller amplitudes [81]. PVEPs may offer an accurate and reliable assessment of residual foveal functions, as patients in advanced stages of RP may have a low percentage of successful or recordable ffERG or PERG signals [81,82,85].

The full-field stimulus threshold (FST) is a simple and fast test that has presented an alternative for measuring the dark-adapted light sensitivity for low-vision patients in recent years. It determines the luminance threshold for detection of a single stimulus flash in a ganzfeld bowl and does not require strict eye fixation [86,87]. FST values represent retinal sensitivity of the most sensitive parts of the still-functioning retina without localization of which areas from which the signal originates. In RP, FST could quantify the wide range of visual impairment representing the range of visual function depending on the early to late stages of disease. FST results also showed strong correlations with full-field ERG amplitude and visual field test results and can provide an alternative to those tests in patients who are unable to undergo a visual field test or have non-detectable ERGs [88].

Overall, electrophysiology testing may be a valuable tool in the early diagnosis and monitoring of RP, as ffERG can identify early characteristic photoreceptor degeneration, even before symptoms are noticeable for patients. This is useful in many cases of early RP and other IRDs in which the retina may appear normal despite clinical symptoms and visual field defects. Furthermore, mfERG and PERG allow an objective evaluation of residual cone function in retinitis pigmentosa patients, which can provide guidance for patient prognosis as it relates to progressive blindness and reduced vision-related quality of life [67]. MfERG, when combined with other techniques such as OCT and VEP, can provide useful information in monitoring macular function in RP [72]. Using a combination of structural imaging and electrophysiological investigations will likely provide better estimations of retinal function in RP patients.

### 2.2. Progressive Cone/Cone-Rod Dystrophy

Inherited cone dystrophies are a heterogeneous group of disorders that affect cone photoreceptor function, with a prevalence estimated at 1 in 40,000 [89]. Cone dystrophies are characterized by visual loss, color vision abnormalities, as well as varying degrees of nystagmus and photophobia [17]. Inherited cone dysfunction syndromes (CODs) usually present in infancy and only impair cone function, with retinal degeneration often restricted to the central retina [90]. Progressive cone-rod dystrophies (CORDs) present in childhood or early adulthood and are characterized by progressive cone photoreceptor loss followed by progressive rod photoreceptor loss. There is significant overlap between progressive cone and cone-rod dystrophies [18].

The inheritance patterns of cone and cone-rod dystrophies include autosomal dominant (most common, 32%), autosomal recessive, and X-linked recessive inheritance [91,92]. Multiple genes have been identified, including *AIPL1*, *CRX*, *GUCA1A*, *GUCY2D*, *PITPNM3*, *PROM1*, *PRPH2/RDS*, *RIMS1*, *SEMA4A*, and *UNC119* [93]. The autosomal dominant mutation of guanylate cyclase activator 1A (*GUCA1A*), which alters the structure and function of guanylyl cyclase-activating protein 1, is associated with both cone and cone-rod dystrophies [94], usually presenting between the third and fifth decade of life with mild photophobia and reduced central vision [17,95].

The fundus exam often reveals a bull’s-eye maculopathy, although some cases may present with minor atrophy and pigmentation of the macular RPE with optic nerve head pallor [92]. On OCT, progressive cone dystrophy shows significant reduction in macular thickness, with atrophy of the outer nuclear layer, in the photoreceptor inner segment/outer segment junction and in the RPE [96]. Higher visual acuity correlated with the maintenance of the photoreceptor inner segment/outer segment junction (IS/OS) layer, thickness of retina, and better preserved foveal structure [96].

Use of electrophysiology for cone dystrophies can be valuable in the early detection and monitoring of possible rod photoreceptor involvement. ffERG of pure cone dystrophies or early CORD shows normal rod responses with abnormal cone signals [97,98]. Cone dystrophy shows normal, preserved rod functions and severe cone dysfunction in ffERG [93]. ffERG can be particularly helpful in early stages when patients are asymptomatic with a normal fundus exam [90]. ERGs on family members with the *GUCA1A* mutation show reduced amplitudes in the 30 Hz flicker and photopic (cones) over scotopic (rod) response, as well as severely reduced bilateral P50 amplitudes on PERG [90,99]. Other ERG findings of cone dystrophy include implicit time delay with the 30 Hz cone flicker responses, delayed a- and b-wave photopic response, as well as decreased amplitudes of both a- and b-waves [90,100]. In CORDs, both rod and cone ERGs are abnormal with predominant cone abnormalities with delayed 30 Hz cone flicker ERG implicit time [17,101].

Overall, electrophysiology testing can be a valuable tool in the early diagnosis and monitoring of progressive cone dystrophy. ERG changes precede the decline in subjective visual functions [96], allowing for early detection. ERGs also can isolate signals of the two major photoreceptor types (rods and cones), which helps distinguish between the spectrum of diseases ranging from COD to CORD [100].

### 2.3. Cone Dystrophy with Supernormal Rod Response

Cone dystrophy with supernormal rod response (CDSRR) is an autosomal recessive disease characterized by cone photoreceptor dysfunction with an abnormal rod ERG response, such as insensitivity to dim light and increased responsiveness to suprathreshold light [102]. Patients typically present with reduced central vision, photophobia, myopia, and color vision abnormalities in the first two decades, with possible nyctalopia later in life [103]. Supernormal rod ERGs are caused by the mutations in the gene *KCNV2*, which encodes a voltage-gated potassium channel subunit expressed in rod and cone photoreceptors. *KCNV2* is also expressed in the heart, and, therefore, CDSRR can be associated with cardiac abnormalities [19,20,21]. There is also a case report of mutations in *PDE6H* that can result in phenotypes similar to CDSRR [104].

The fundus appearance may be normal in the early stage with a supernormal rod ERG, but macular pigment disturbance and atrophy can be presented later. OCT shows variable characteristics, including discontinuous IS/OS junction, foveal gap and central retinal thinning [105].

The ERGs of cone dystrophy with supernormal rod ERG are pathognomonic and diagnostic of the disorder. Like other cone dystrophy disorders, the light-adapted (LA) 3.0 and LA 3.0 30 Hz ffERGs show significant reduction and delay in those patients [106]. PERG is always abnormal in the patients with supernormal rod ERG and can be undetectable in severe macular dysfunction, although some other cone and cone-rods dystrophies may have relatively preserved macular function and PERGs in early disease [107]. Several pathognomonic findings of this disorder occur in the rod system: (1) In response to a very dim white flash (0.002 phot cd s/m^2^), there is no detectable ERG response. (2) In response to a standard dark-adapted dim flash (DA 0.01), the ERG shows significant delay of implicit time and subnormal amplitude. (3) From 0.01 phot cd s/m^2^, b-wave amplitude increases rapidly with small increases in stimulus intensity. (4) In response to DA 10.0 ERG, a-wave shows a normal slope (normal phototransduction) at the beginning, followed by a broadened shape with smaller slope (a late negative component) [106,108].

Overall, electrophysiology testing is the primary tool in the diagnosis of CDSRR due to its pathognomonic and characteristic findings. To further work up this suspected diagnosis, genetic testing is necessary to confirm the mutations in either the *KCNV2* or *PDE6H* genes.

### 2.4. Enhanced S-Cone Syndrome

Enhanced S-cone syndrome (ESCS) is a rare, slowly progressive retinal dystrophy characterized by nyctalopia from the first decade of life, reduced visual acuity, hyperopia, and no nystagmus [109,110]. ESCS is an autosomal recessive disorder related to mutations in *NR2E3*, which encodes a photoreceptor nuclear receptor transcription factor critical for rod photoreceptor development, leading to excess S (blue) cones at the expense of other photoreceptor subtypes [111]. The disease is characterized by an abnormal ratio of S (blue) to L/M (red/green) cone function, the absence of rods, and progressive retinal degeneration [112]. In addition to the variety of mutations in the NR2E3 gene that causes the autosomal recessive ESCS, autosomal dominant mutations in NR2E3 have been found to cause retinitis pigmentosa, although the distinct inheritance patterns and electrophysiological findings can help differentiate these conditions [22,23].

On fundus examination, there are characteristic deep nummular clumped pigmentary lesions around the vascular arcades with possible yellow streaks, as well as macular retinoschisis and cystoid maculopathy [113]. On OCT, there is a significantly thickened outer nuclear layer as well as macular abnormalities including foveomacular schisis and macular holes [114].

**Figure 3:**
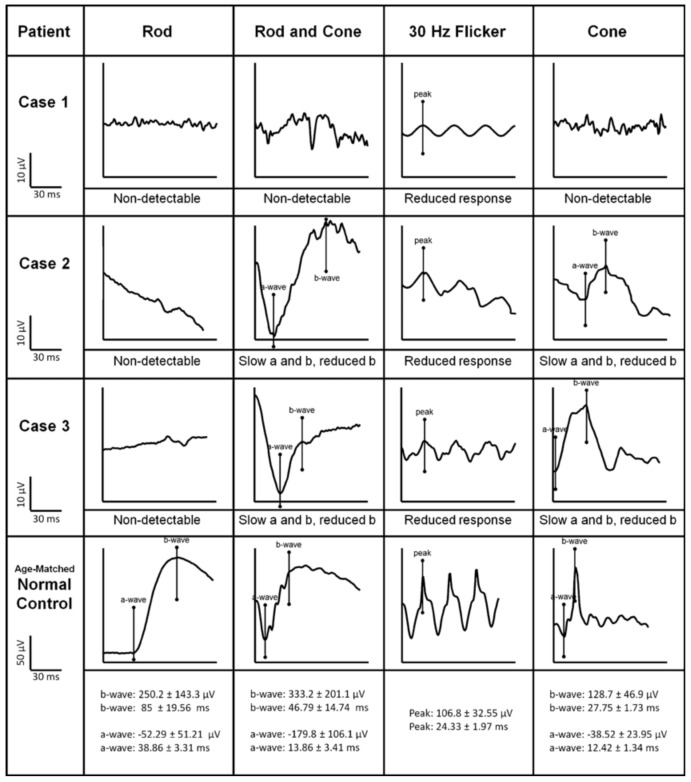
ISCEV-standard rod-specific, maximal mixed rod and cone, 30 Hz flicker stimulus, and transient single-cone ERG waveforms are shown. The scales, with normal ranges for each condition, are listed in the bottom row (reused from Park et al., 2013 with permission [119]).

The abnormality of ERGs in ESCS is also pathognomonic of the disorder. ISCEV-standard recordings of ffERG in an ESCS patient are shown in Figure 3. ffERG dark-adapted 0.01, which is rod-specific, is almost always undetectable due to the absence of rods [115]. DA 3.0 ERG (mixed rod and cone response) is delayed and usually reduced [111]. The 30 Hz flicker ERG shows characteristically delayed implicit time and reduced amplitude [116]. S-cone specific and ON-/OFF ERGs show supernormal, higher amplitudes with simplified waveforms and delayed peak, which is pathognomonic of the disorder [117]. The pattern ERG, if detectable, usually shows a delayed and reduced P50 component [117,118]. MfERG shows preservation of central responses but delayed and reduced responses in peripheral rings [117].

Overall, electrophysiological testing is an invaluable tool in the diagnosis of ESCS, which shows pathognomonic ERG findings and identifies patients for targeted specific molecular screening to finally confirm the diagnosis [109].

### 2.5. Bradyopsia

Bradyopsia, or “slow vision”, is a rare retinal dysfunction syndrome characterized by delayed dark to light adaptation, photophobia, difficulty in tracking moving objects, normal color vision, normal fundus appearance and mildly decreased visual acuity from early childhood [120,121]. Bradyopsia is caused by genetic defects in *RGS9* or *R9AP*, which are associated with defects in RGS9 or its anchor protein R9AP. RGS9/R9AP have a critical role in the rate of recovery from phototransduction by deactivating transducin in rod and cone phototransduction cascades [24].

The ERG of bradyopsia shows diagnostic findings of the disorder but also requires testing of cone function under dark adaptation and understanding of impaired recovery after a bright flash for correct diagnosis, which needs a protocol in addition to the current ISCEV standard protocols [108]. DA 0.01 (rod-specific) ERG and DA 3.0 ERG are normal or mildly reduced, while LA 3.0 ERG is subnormal. The pattern ERG and standard LA 3.0 30 Hz flicker ERG are undetectable in all patients with the *RGS9* mutation [120]. Additionally, there are important findings of the disorder: (1) a scotopic (rod) red flash ERG under dark adaptation evokes normal rod and dark-adapted cone function (but severely abnormal cone function under photopic conditions); (2) dim flicker stimulus under dark adaptation reveals normal ERG response at the beginning of stimulation, which attenuates to undetectable response after 2 s; (3) DA 10.0 ERG reveals normal response to the first bright flash, and the subsequent responses to further flashes are attenuated, which takes up to 2 min to recover [24,120]. Similarly, ERG in the mouse model lacking *RGS9* shows a normal response in the first flash with reduction in subsequent flashes and prolonged recovery [25,122].

Overall, in the diagnosis of bradyopsia, electrophysiological testing is a valuable tool that can identify characteristic ERG findings. Furthermore, additional ERG protocols can characterize the functional deficiencies.

### 2.6. Bietti Crystalline Dystrophy

Bietti crystalline dystrophy (BCD) is a rare chorioretinal dystrophy characterized by multiple, intraretinal crystalline lipid deposits with progressive chorioretinal atrophy commencing at the posterior pole that can eventually extend beyond the macula [27]. The clinical features of the disease include central vision impairment, nyctalopia, reduced visual acuity and constriction of visual fields that progress between the 2nd and 4th decades of life, with higher prevalence in the East Asian population [123,124]. BCD is an autosomal recessive disease characterized by mutations in the *CYP4V2* gene, a major cytochrome P450, which encodes the *CYP4V2* protein found in epithelial cells of the retina and cornea [26].

On fundus examination, there are intraretinal crystals, as well as atrophy of the retinal pigment epithelium and choriocapillaris complex, that are initially localized to the posterior periphery and later the central fundus [28]. As the disease progresses, chorioretinal atrophy expands centrifugally, and the crystals diminish in number. On OCT, intraretinal crystals appear as hyperreflective dots present in almost all retinal layers that are accompanied by attenuated areas with hyper-reflective borders called outer retinal tubulations (ORT) [125,126,127,128]. ORTs, typically seen in the outer nuclear retinal layers, are likely due to rapid and severe photoreceptor degeneration and rearrangement [127]. The degeneration in BCD seen by OCT is most prominent in the outer retina, including the photoreceptor layer.

Electrophysiology testing is more useful in assessing the extent of the disease rather than as an initial diagnostic tool in BCD. The ffERG shows varying degrees of rod and cone dysfunction, ranging from normal to reduced or undetectable amplitudes of scotopic and photopic a- and b-wave responses [129,130,131]. The ffERG implicit times are also prolonged in BCD, especially in scotopic ERG, which correlates with the clinical presentation of nyctalopia [132]. The progression of the disease may follow a rod-cone dystrophy pattern according to ffERG findings. Some studies show the correlation between ffERG and disease severity, in which the patients with RPE atrophy and intraretinal crystals confined to the posterior pole show normal or mildly affected ffERG [129]. The ffERG is more likely to be abnormal in the later course of the disease when the peripheral visual field is severely affected [28], although the ffERG can also remain normal in some patients in later stages of the disease, even with severe RPE and choroid atrophy [133].

In BCD, mfERG could be helpful in detecting focal regions of abnormal retinal function, especially in the phenotypes that predominantly affect the posterior pole with normal ffERG. Significant reductions in N1 and P1 amplitudes as well as delays in P1 (and to a lesser degree in N1) implicit times of mfERG can be seen in BCD patients [132,134], with relatively preserved peripheral responses [132,135]. Because a BCD patient with normal peripheral retina may have normal ffERG but abnormal mfERG [136], mfERG is more sensitive than ffERG in identifying the defective areas of the retina, which can be helpful in the evaluation and follow-up of these patients [132].

Overall, electrophysiological testing may not be critical in the initial diagnosis of BCD. However, it can be used to monitor the progression of retinal degeneration in BCD over time.

### 2.7. Late-Onset Retinal Degeneration (L-ORD)

Late-onset retinal degeneration (L-ORD) is a rare retinal dystrophy characterized by progressive dark-adaptation abnormalities starting in the 4th and 5th decade of life and bilateral loss of vision in the 6th and 7th decades [137], which should be differentiated from age-related macular degeneration in term of the relatively earlier onset, greater extent of disease, pace of progression, and presence of nyctalopia in L-ORD. Additionally, features such as anterior segment involvement, including notably long anterior zonules and iris atrophy, can help in distinguishing L-ORD from other inherited or age-related disorders [137]. It is a fully penetrant autosomal dominant condition relating to variants in the *C1QTNF5* gene, which normally encodes a protein involved in cellular adhesion, extracellular matrix modification and in AMP-activated protein kinase signaling [29]. The mutation is thought to disrupt RPE adhesion and increase build-up of lipid-rich material between RPE cells and Bruch membrane in aging patients with L-ORD [30].

On fundus examination, there are yellow-white, lipid-rich drusen subretinal deposits in the pericentral and midperipheral retina [138,139]. Scalloped chorioretinal atrophy that spreads from the temporal retina towards the fovea appears later in the disease course [140]. In the 7th decade of life, the fovea may be compromised due to the progressive atrophy and choroidal neovascularization [141]. On OCT, there are sub-RPE deposits with disturbance of the IS/OS junction and outer nuclear layers, with hyper-reflective deposits, thickening of the RPE/Bruch’s membrane complex, and diffuse choroidal thinning suggesting loss of choriocapillaris [142].

Use of electrophysiology for L-ORD can be helpful in some cases to identify abnormalities in early disease [137,138,142], which often shows a generalized rod-cone pattern of dysfunction that appears to be more progressive at later stages of the disease [140,143]. In L-ORD patients, significant reduction of scotopic ERG is usually observed [144], while the photopic ffERG is affected in the later stage [143]. There is correlation between the decreased ffERG response to areas of drusenoid deposits and atrophy [30,138,140,143,145], because ffERG can still be elicited from the unaffected retinal areas [145].

In a study of L-ORD with ffERG, there was significant improvement in b-wave under DA 0.01 and a-wave under DA 3.0 after a prolonged dark adaptation of 16 h, suggesting an opportunity for treatment in patients with moderate to advanced L-ORD. It suggests the possibility of partial reversion of rod dysfunction [145].

In L-ORD, pattern ERG shows that there is also severe macular involvement in addition to rod-cone dysfunction on ffERG [142]. Therefore, a severe macular involvement can be detectable using PERG in L-ORD patients. MfERG shows reduced amplitudes in the areas of drusenoids, corresponding to areas of reduced sensitivity on microperimetry maps [146]. In three L-ORD patients, EOG showed a normal light peak/dark trough (Arden) ratio, with two cases demonstrating abnormal dark trough amplitude and delayed light peak [143].

Overall, electrophysiological testing may not be a critical diagnostic tool for L-ORD because ffERG can be normal in some patients, especially in early disease [147]. However, electrophysiological testing may be the objective complementary tool to monitor the progression of retinal degeneration as well as to identify partial reversion of rod dysfunction with extended dark adaptation in L-ORD.

### 2.8. Fundus Albipunctatus

Fundus albipunctatus (FA) is a type of congenital stationary night blindness characterized by early onset and nonprogressive nyctalopia. FA is an autosomal dominant or recessive disorder caused by either a homozygous or compound heterozygous mutation in the *RDH5* gene that encodes 11-*cis*-retinol dehydrogenase, which converts 11-*cis*-retinol into 11-*cis*-retinal in the retinal pigment epithelium. This leads to delayed regeneration of visual pigment, and mutations of the *RDH5* gene can cause a progressive cone dystrophy as well as night blindness [32,148].

Fundus examination shows numerous dull-white punctate lesions scattered throughout the fundus without autofluorescence, indicating a reduced supply of 11-*cis*-retinal to the photoreceptors [149,150].

Use of electrophysiology for fundus albipunctatus can be valuable and reliable in diagnosis and help differentiate patients who may also additionally have cone dystrophies. Most patients with fundus albipunctatus have a nonprogressive rod dysfunction without retinal vessel attenuation, or pigment clumping in the retina, which is similar to retinitis functata albescens in Section 2.9. However, other patients with fundus albipunctatus may have cone dystrophy, leading to loss of central visual acuity and reduced cone ERG amplitudes [32]. Standard scotopic ffERG shows moderate to severe generalized rod system dysfunction. Scotopic dim flash ERGs can be undetectable or subnormal but can be normalized after prolonged dark adaptation. Scotopic bright flash ERG shows a reduced maximal response. The use of a red stimulus under dark adaptation and extended recordings in the dark-adapted state suggest dark-adapted cones as the probable source of the ERG signals. An improvement in the dark-adapted ERG response is considered a feature of FA because of the slow rate of regeneration of photopigment but can also be found in retinitis punctata albescens patients [151]. Photopic responses may have subnormal amplitudes [151,152,153].

Overall, electrophysiology testing, together with genetic testing, is the most reliable tool in the diagnosis of FA. Additional retinal imaging (fundus photographs, fundus autofluorescence, and OCT) can show characteristic findings and may aid with the diagnosis. Particularly in younger patients with characteristic features of fundus albipunctatus, cone and rod function can revert to normal after a prolonged period of dark adaptation.

### 2.9. Retinitis Punctata Albescens

Retinitis punctata albescens (RPA) is a rare progressive retinal cone-rod dystrophy and considered to be an atypical variant of retinitis pigmentosa. It characteristically causes nyctalopia and is found at a prevalence as high as 1 per 4500 population in northern Sweden [154]. It can be caused by autosomal recessive mutations in the gene encoding cellular retinaldehyde–binding protein 1 (*RLBP1*), which is involved in retinal metabolism of vitamin A [33,155,156], autosomal dominant mutations in peripherin/*RDS* [34], or rhodopsin gene [35].

Fundus exam shows small yellow-white deposits throughout the retina and peripheral areas of atrophy at the level of the retinal pigment epithelium [155]. Several features of RPA such as progression to narrow vasculature, pigmentary degeneration, and field loss can distinguish this diagnosis from fundus albipunctatus, which can have similar white dots in the fundus.

Utilizing electrophysiology for retinitis punctata albescens can be helpful in the diagnosis of the disease. ffERG performed after standard dark adaptation shows moderate to severe generalized rod system dysfunction. The dark-adapted isolated rod ERG response is severely reduced or undetectable [157]. MfERG may show a reduced cone response in the central region of the tested area with no improvement after 20–24 h of dark adaptation [158].

Overall, electrophysiology testing can be helpful in distinguishing RPA from fundus albipunctatus, which most often shows stationary rod receptor dysfunction and recovery of cone and rod function after a prolonged period of dark adaptation that is not often seen in RPA. Genetic screening, as well as the presence of progressive loss of photoreceptor cell function, attenuated retinal vessels and pigmentary clumping, are also the important clinical manifestations of RPA in a diagnostic workup to exclude fundus albipunctatus. A summary of the electrophysiological findings of RPA and other non-macular inherited retinal dystrophies are highlighted in Table 2.

## 3. Conclusions

Electrophysiological testing is an important tool for the accurate diagnosis and prognosis of inherited retinal dystrophies. Many non-macular inherited retinal dystrophies affect the a- and b-wave amplitudes and implicit times of ffERG, as well as the parameters in mfERG, PERG, EOG, and other electrophysiological tests. Some disorders, such as cone dystrophy with supernormal rod ERG, enhanced S-cone syndrome, and bradyopsia, show pathognomonic findings in ffERG, making electroretinography vital to the diagnosis of the disease. Overall, electrophysiological findings enhance the clinical and genetic characterization of inherited retinal diseases.

## Figures and Tables

**Table 1:** Genetic information of the retinal dystrophies reviewed in this study.

Name	OMIM	Gene	Inheritance	References
Retinitis Pigmentosa	268000 (heterogeneous)	*RHO*, *PRPF31*, *RP1*, *RPGR*, *RP2*, *PDE6*, etc.	Autosomal dominant, X-linked, autosomal recessive	[14,15,16]
Cone Dystrophy	120970, 602093, etc. (heterogeneous)	*AIPL1*, *CRX*, *GUCA1A*, *GUCY2D*, *PITPNM3*, *PROM1, PRPH2/RDS*, *RIMS1*, *SEMA4A*, and *UNC119*	Autosomal dominant, X-linked, autosomal recessive	[17,18]
Cone Dystrophy with Supernormal Rod ERG	610356	*KCNV2*	Autosomal recessive	[19,20,21]
Enhanced S-Cone Syndrome	268100	*NR2E3*	Autosomal recessive	[22,23]
Bradyopsia	608415 or 620344	*RGS9* or *R9AP*	Autosomal recessive	[24,25]
Bietti Crystalline Dystrophy	210370	*CYP4V2*	Autosomal recessive	[26,27,28]
Late-Onset Retinal Degeneration	605670	*C1QTNF5*	Autosomal dominant	[29,30]
Fundus Albipunctatus	601617	*RDH5*	Autosomal recessive or dominant	[31,32]
Retinitis Punctata Albescens	136880	*RLBP1*, *RHO*, *PRPH2*	Autosomal recessive or dominant	[33,34,35]

**Table 2:** Electrophysiology findings in inherited retinal dystrophies.

Name	ffERG	mfERG	Pathognomonic Findings	References
Retinitis Pigmentosa	A−, I−	A−, I−	No	[65,66,68,69,70,71]
Cone/Cone-Rod Dystrophy	A−, I−		No	[97,98]
Cone Dystrophy with Supernormal Rod ERG	A− (except supernormal S-cone ERG), I−		Yes (see Section 2.3)	[106,107,108]
Enhanced S-Cone Syndrome	A−, I−		Yes (see Section 2.4)	[115,116,117,118]
Bradyopsia	A−		Yes (see Section 2.5)	[24,25,120,122]
Bietti Crystalline Dystrophy	A−, I−	A−, I−	No	[126,127,128]
Late-Onset Retinal Degeneration	A−	A−	No	[30,138,140,143,145]
Fundus Albipunctatus	A−		No	[151,152,153]
Retinitis Punctata Albescens	A−	A−	No	[157,158]

Abbreviations: A, Amplitude or LP:DT ratio; I, implicit time.

## Data Availability

Not applicable.
